# HSDNet: a poultry farming model based on few-shot semantic segmentation addressing non-smooth and unbalanced convergence

**DOI:** 10.7717/peerj-cs.2080

**Published:** 2024-06-07

**Authors:** Daixian Liu, Bingli Wang, Linhui Peng, Han Wang, Yijuan Wang, Yonghao Pan

**Affiliations:** College of Information Engineering, Sichuan Agricultural University, Ya’an, China

**Keywords:** Few-shot learning, Smooth loss, Semantic segmentation

## Abstract

Poultry farming is an indispensable part of global agriculture, playing a crucial role in food safety and economic development. Managing and preventing diseases is a vital task in the poultry industry, where semantic segmentation technology can significantly enhance the efficiency of traditional manual monitoring methods. Furthermore, traditional semantic segmentation has achieved excellent results on extensively manually annotated datasets, facilitating real-time monitoring of poultry. Nonetheless, the model encounters limitations when exposed to new environments, diverse breeding varieties, or varying growth stages within the same species, necessitating extensive data retraining. Overreliance on large datasets results in higher costs for manual annotations and deployment delays, thus hindering practical applicability. To address this issue, our study introduces HSDNet, an innovative semantic segmentation model based on few-shot learning, for monitoring poultry farms. The HSDNet model adeptly adjusts to new settings or species with a single image input while maintaining substantial accuracy. In the specific context of poultry breeding, characterized by small congregating animals and the inherent complexities of agricultural environments, issues of non-smooth losses arise, potentially compromising accuracy. HSDNet incorporates a Sharpness-Aware Minimization (SAM) strategy to counteract these challenges. Furthermore, by considering the effects of imbalanced loss on convergence, HSDNet mitigates the overfitting issue induced by few-shot learning. Empirical findings underscore HSDNet’s proficiency in poultry breeding settings, exhibiting a significant 72.89% semantic segmentation accuracy on single images, which is higher than SOTA’s 68.85%.

## Introduction

In recent years, the rapid advancement of computer science technologies, coupled with the growing demand for food due to population increase, has propelled smart agriculture into a focal point of research and practice. The application of embedded devices and Internet of Things (IoT) technology has not only facilitated the digitization and visualization of agricultural production processes but has also significantly enhanced the level of automation in agriculture. This technological transformation has markedly improved agricultural productivity and provided farmers with an abundance of useful information and tools, optimizing their management and decision-making processes (Kassim, 2020; Prathibha, Hongal & Jyothi, 2017; Gondchawar & Kawitkar, 2016; Lipper et al., 2014). However, to further advance the automation in intelligent farming, the capabilities of artificial intelligence (AI) must be leveraged (Shaikh et al., 2021). Poultry farming, a crucial component of agricultural production and a major source of meat and egg products is integral to the evolution of smart agriculture. Particularly in the realms of real-time monitoring and production management, the application of AI can significantly enhance efficiency and yield.

Moreover, due to the advancements in hardware technology and substantial increases in computational power, artificial intelligence has embarked on a pivotal phase of development. Specifically, the introduction of Deep Residual Networks (ResNet), which adeptly mitigated the degradation issue of deep learning models, has catalyzed rapid advancements in the field of computer vision. Subsequent progress in various visual tasks, such as object detection, pose estimation, and semantic segmentation, has not only facilitated the widespread application of technologies like autonomous driving and facial recognition but has also enabled the application of advanced techniques like remote sensing segmentation in diverse fields such as agriculture, urban planning, and environmental monitoring, thereby leading to numerous technological breakthroughs and industrial upgrades (He et al., 2016; Padilla, Netto & Da Silva, 2020; Zou et al., 2023; Wang et al., 2018). Recent advancements in technologies such as Neural Radiance Fields (NeRF) and Stable Diffusion have notably propelled progress in 3D modeling and image generation. NeRF provides a methodology for modeling 3D scenes from 2D image sets, while Stable Diffusion opens new pathways for image generation (Mildenhall et al., 2021; Rombach et al., 2022). Additionally, the advent of the Transformer architecture has significantly accelerated developments in natural language processing (NLP), spawning large language models like ChatGPT, which assist individuals in their daily tasks (Vaswani et al., 2017; Ouyang et al., 2022).

Despite the rapid development of artificial intelligence, its application in smart agriculture predominantly remains within the realm of research. For instance, Li et al. (2022a) have utilized target detection technology for carp identification, significantly reducing costs compared to traditional embedded chip methods. Similarly, Li et al. (2022b) employed instance segmentation for intelligent monitoring of geese, testing in real-time on embedded devices to ensure feasibility for large-scale deployment. Jiang et al. (2022) used target detection technology to identify the sex of ducks and improve returns. Moreover, Zhang et al. (2019) have successfully implemented automatic detection and tracking of individual pigs in piggeries. Traditional poultry farming grapples with challenges such as early disease detection, optimization of stocking density, and behavioral monitoring, often hindered by the lack of precise and real-time monitoring mechanisms. The application of semantic segmentation technology enables the accurate identification and tracking of individual animals within a poultry flock (Okinda et al., 2020). This approach facilitates the automatic differentiation of each poultry, monitoring their range of activity and behavioral patterns.

While substantial progress has been made in integrating intelligent processes into poultry farming, the practical application of these technologies in complex agricultural environments presents ongoing challenges. Current studies often focus on a single animal species in specific settings, limiting the broader applicability of these technologies. Taking China’s chicken breeding industry as an example, there are over twenty breeds in commercial farming alone, not to mention various small-scale breeds and hybrids, necessitating higher adaptability of the models. Present AI techniques, particularly in computer vision, predominantly rely on supervised learning, requiring extensive labeled data to train efficient models, which is both time-consuming and costly in practice (Jaiswal et al., 2020; Yang et al., 2022b). More crucially, due to inherent differences in breeding environments, models may yield varying recognition results in different contexts, a phenomenon known in machine learning as ‘domain shift’ or ‘domain adaptation’. Specifically, many real-world factors such as weather conditions, agricultural breeds, and minor variations in breeding environments can affect the actual performance of models, even if they are trained on specific datasets (Ganin & Lempitsky, 2015; Kang et al., 2019; Ye et al., 2022).

To tackle the challenges encountered in practical applications, this article integrates few-shot learning into the fusion of AI and poultry farming. This strategy effectively mitigates issues related to performance degradation due to variations in breeds and environments. Traditional deep learning methods typically require extensive data for model training, which is not always feasible in diverse agricultural settings. Few-shot learning, capable of performing effective detection or segmentation tasks with just 1–5 images, reduces the workload of data collection and annotation, avoids accuracy loss due to limited data, and enhances the model’s generalizability and practicality (Sung et al., 2018).

In this article, our focus is on three primary poultry species: chickens, ducks, and geese. To demonstrate the robustness of the HSDNet model, we have also included the golden crucian carp, a species from a completely different environment, as part of the HSDNet model’s robustness testing. Compared to other application domains, poultry environments possess unique characteristics (Lin et al., 2014; Cordts et al., 2016). In most general few-shot segmentation scenarios, dataset backgrounds are typically urban or other simplified settings. However, poultry farming’s complex background, comprising factors like soil, vegetation, and weather, coupled with high-density livestock clustering, poses substantial challenges to segmentation tasks. The intricate and dense nature of the poultry environment often results in non-smooth losses, complicating model convergence during training. To address this issue, we introduce the Sharpness-Aware Minimization strategy specifically optimized for the non-smoothness of losses, helping the model converge more stably in complex agricultural backgrounds, thereby enhancing the overall performance and accuracy of the model.

In free-range environments, poultry typically exhibit a sparse distribution, with partial clustering around feeding areas. This leads to a strong imbalance between positive and negative samples, affecting model convergence. In few-shot learning, limited training data can easily lead to overfitting, exacerbating the imbalance caused by the distribution of poultry farming. To mitigate this problem, we introduce Dice loss, which alleviates the imbalance loss caused by insufficient samples in few-shot learning.

Summarizing, the contributions of this article are as follows:

- We pioneered the integration of few-shot learning into artificial intelligence applications for poultry farming with the introduction of HSDNet, a semantic segmentation model customized for poultry. This innovation advances the practical applicability of models in the livestock industry.
- To address the issue of non-smooth losses in agricultural scenarios affecting model accuracy, we introduce the Sharpness-Aware Minimization strategy.
- By employing Dice loss, we successfully reduce the overfitting problem caused by the strong imbalance between positive and negative samples during model training.

The structure of this article is as follows: “Introduction” provides the introduction, “Related Work” discusses related work, “Methods” elaborates on the model, “Dataset” describes dataset generation, “Experiments” presents the experiments, “Discussion” offers a discussion of the results, and “Conclusion” concludes the article.

## Related Work

### Artificial intelligence and smart agriculture

The amalgamation of artificial intelligence (AI) within the agricultural sector, particularly in the domain of smart farming, is revolutionizing the way we approach agricultural productivity and sustainability. AI technologies, including machine learning, computer vision, and semantic segmentation, are increasingly being applied to enhance various aspects of farming practices.

In the sphere of smart agriculture, the fusion of advanced technologies has led to significant advancements in farm management and crop health monitoring. Notably, The amalgamation of remote sensing technology with deep learning has heralded new frontiers in the monitoring of farmland health (Shafi et al., 2020). Machine learning algorithms are being utilized to foresee crop diseases and pest infestations, empowering farmers to enact timely preventative strategies (Ouhami et al., 2021). Furthermore, Razfar et al. (2022) have developed a computer vision-based weed detection system capable of efficiently identifying weeds within soybean plantations, thus enhancing crop yields.

Artificial intelligence has proven to be a game-changer in the field of poultry farming. Hossain et al. (2023) developed an AI-assisted automated system for the early detection of chicken diseases from fecal images captured by smartphones, thus enabling prompt disease identification and mitigating poultry losses. Yao et al. (2023) applied object detection technology to determine the age of pigeons, allowing for the rapid and accurate recognition of each pigeon’s growth stage in a cage, enabling on-demand feeding strategies that reduce costs. Additionally, Qiao, Truman & Sukkarieh (2019) utilized the Mask R-CNN deep learning framework for automatic segmentation and contour extraction of cattle, facilitating the acquisition of real-time information on individual cattle. However, this approach requires extensive training with high-quality images of key cattle, which presents a scalability challenge for the model.

### Few-shot semantic segmentation

Few-shot learning, particularly in the realm of semantic segmentation tasks, has been a focal point of research in the computer vision field due to its substantial potential. Traditional deep-learning methods often require a large volume of labeled data to train models. However, obtaining extensive annotated data is often impractical in real-world applications. Consequently, few-shot learning methods, especially those requiring only a minimal amount of labeled data, have emerged as a research hotspot.

Wang et al. (2024b) unveiled an innovative approach that harnesses language cues through a vision-language-driven mask distillation scheme, combining a vision-language pretraining model and a mask refiner to generate high-quality pseudo-semantic masks from textual prompts. This method is further refined by integrating distributed prototype supervision and a complementary correlation matching module for enhanced semantic clarity. Li, Chen & Xiong (2024) introduced a nuanced dual prototype learning framework, employing a second-order prototype (SOP) to grasp higher-order statistical insights alongside a self-support first-order prototype with a constraint mechanism (SSFPC), significantly boosting the model’s adaptability. Wang et al. (2024a) crafted an Adaptive FSS framework featuring the Prototype Adaptive Module (PAM), specifically designed to amplify class-specific details by leveraging precise category information from the support set. Chang et al. (2024) devised a feature disentanglement and recombination network (DRNet), utilizing self-attention and cross-attention for meticulous foreground feature alignment, with prototypes derived from these features guiding the segmentation process. This is augmented by a strategic joint learning approach to ensure accurate segmentation of both familiar and novel objects. Peng et al. (2023) delve into pixel-based support associations using transformer architecture, enhancing the segmentation precision from coarse to fine granularity through a dedicated matching module and relationship distillation. These pioneering efforts significantly propel the field of few-shot semantic segmentation towards greater versatility and precision.

In the agricultural sector, Few-Shot Semantic Segmentation (FSS) is set to transform traditional farming methods by enabling precise identification of crops and diseases with minimal labeled data. Tan, Chen & Yan (2023) introduced a novel diffusion model called DifFSS, tailored for agriculture’s FSS needs. It leverages diffusion models’ generative power to enhance segmentation accuracy for various crops and conditions without changing the base network structure. Concurrently, Yang et al. (2022a) tackled plant disease segmentation by proposing an FSS model that uses a multi-scale and multi-prototype matching approach. Additionally, Maranelli (2022) concentrated on effective weed control in smart farming, applying the FSS framework to address the challenge of scarce annotated data. Their method, which optimizes image embeddings and investigates the impact of core model parameters on segmentation results, includes ensemble techniques to significantly improve weed detection in agricultural datasets.

## Methods

The animal use protocol listed below has been reviewed and approved by the Sichuan Agricultural University Animal Ethical and Welfare Committee, with approval number 20230179.

### HSDNet network architecture

**Figure 1:**
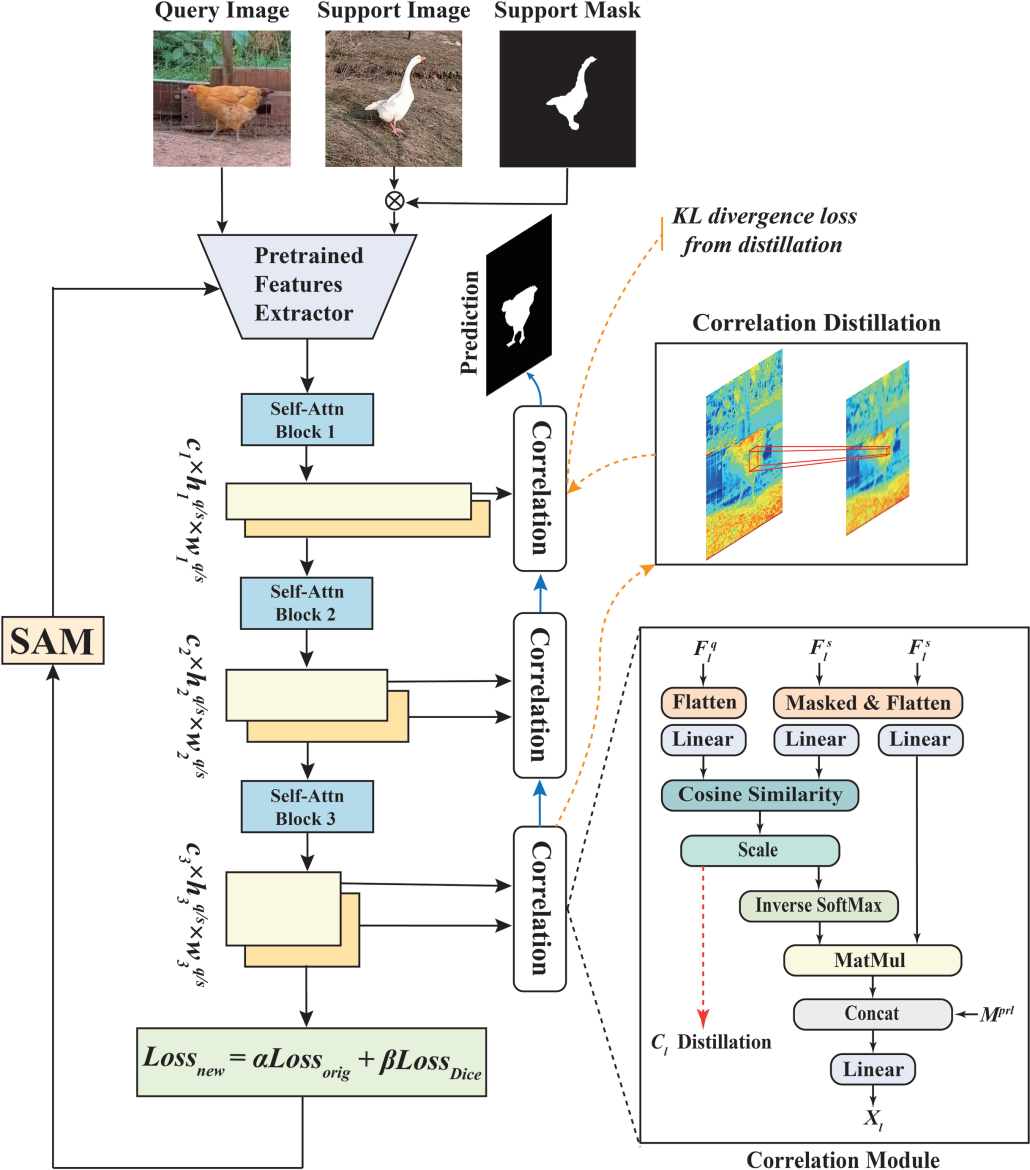
This diagram intricately illustrates the network structure of HSDNet. Within this architecture, we have incorporated the Sharpness-Aware Minimization strategy and added Dice loss to better accommodate the experimental requirements and application scenarios in poultry farming. Figure source credits: https://github.com/DaixianLiu/DaixianLiu.github.io/tree/main/chicken, https://github.com/DaixianLiu/DaixianLiu.github.io/tree/main/goose.

HSDNet is an innovative advancement based on the Hierarchically Decoupling Matching Network (HDMNet) (Peng et al., 2023), meticulously crafted to better align with the specific needs of poultry farming. The network architecture of HSDNet is illustrated in Fig. 1.

HDMNet was chosen as the foundational model for its groundbreaking design, which effectively overcomes the constraints of conventional approaches in semantic feature and prototype representation. Built upon the Transformer architecture (Vaswani et al., 2017), originally applied in natural language processing, the Transformer’s cornerstone is its self-attention mechanism. This mechanism allows the model to allocate different attention weights to each element when processing sequences, employing multiple self-attention layers to process data in parallel. Such parallel processing enables the model to concurrently capture diverse facets or features of the input data, enhancing its efficiency. The incorporation of the Transformer architecture in few-shot semantic segmentation transcends the limitations of semantic-level prototypes and capitalizes on pixel-wise alignment.

HDMNet introduces a novel hierarchical matching structure, strategically decoupling the downsampling and matching processes and utilizing independent self-attention layers to construct hierarchical features meticulously. This design ensures the preservation of sequence feature purity and maintains consistency in pattern matching.

During the decoupling process, the extracted query and support features from the backbone are individually channeled into sequential transformer blocks, composed exclusively of self-attention layers. Notably, the downsampling layer is strategically positioned between blocks, forming a hierarchical structure that potentially enhances interscale correlation.

Transitioning to the technical intricacies, let’s delve into the intermediate feature maps of *L* stages, denoted as $\left\{ {F_l^q} \right\}_{l = 1}^L$ and $\left\{ {F_l^s} \right\}_{l = 1}^L$. For simplicity, let’s assume that $\left\{ {F_l^q} \right\}$ and $\left\{ {F_l^s} \right\}$ maintain the same spatial size $\left[ {{c_l} \times h_l^{q/s} \times w_l^{q/s}} \right]$.

$$h_l^{q/s} = {{{H^{q/s}}} \over {{2^{l + 2}}}},\quad w_l^{q/s} = {{{W^{q/s}}} \over {{2^{l + 2}}}},$$

$l$ is the stage index, and ${c_l}$ denotes the feature channel number. Finally, $\left\{ {F_l^q} \right\}_{l = 1}^L$ and $\left\{ {F_l^s} \right\}_{l = 1}^L$ are used to yield correlations $\left\{ {C_l} \in {\mathbb{R}}{{}^{h_l^qw_l^q \times h_l^sw_l^s}} \right\}_{l = 1}^L$ and enriched query features $\left\{ {A_l} \in {\mathbb{R}}{{}^{{c_l} \times h_l^q \times w_l^q}} \right\}_{l = 1}^L$.

HDMNet integrates a decoder transitioning from coarse to fine granularity. The coarse-grained feature ${\mathrm{A}}_{l + 1}^\prime$ is resized to match the spatial dimensions of the fine-grained feature ${\mathrm{A}}_l^\prime$. Subsequently, they are fused using an MLP layer and residual connections, denoted as

(1) $${\mathrm{A}}_l^\prime = ReLU\left( {MLP\left( {{A_l} + {\zeta _l}\left( {A_{l + 1}^\prime } \right)} \right)} \right) + {\zeta _l}\left( {A_{l + 1}^\prime } \right)$$

Diving deeper into the mathematical representation, let’s consider that $l$ signifies the hierarchical stage, and ${\zeta _l}:{\mathbb{R}}{{}^{H \times W}} \;\mapsto\; {\mathbb{R}}{{}^{{h_l} \times {w_l}}}$ represents the bilinear-interpolation resize function, which adjusts the input size to match that of the output. Subsequently, a convolution layer with a $1 \times 1$ kernel size is applied to $A_1^\prime$, followed by a bilinear up-sampling layer, to predict the query mask ${M^{out}} \in {\mathbb{R}}{{}^{H \times W}}$.

**Figure 2:**
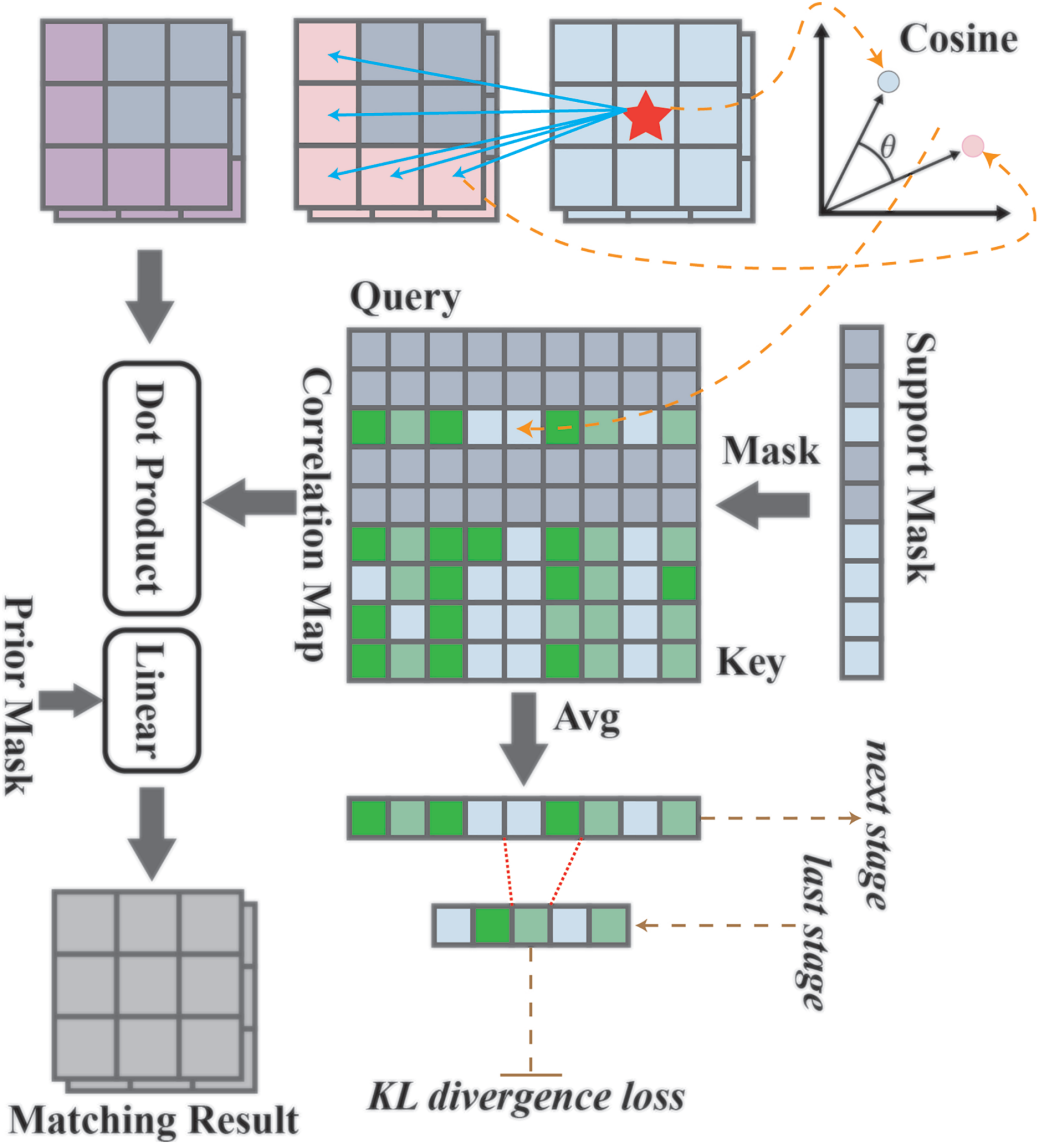
The matching module utilizes correlation mechanisms and distillation techniques to achieve more precise feature matching and information extraction. Data sourced from the matching module.

The HDMNet, within its hierarchical paradigm, strategically decouples the feature parsing and matching processes and introduces an innovative matching module, illustrated in Fig. 2. This module operates based on a correlation mechanism, identifying regions with peak correlation and cosine similarity, and subsequently fusing them with the generated high-order prior masks. Initially, the input feature is transformed according to

(2) $$\matrix{ {{{\hat F}^q} = \varphi \left( {{F^q}} \right),}  \cr  {{{\hat F}^s} = \varphi \left( {{F^s} \odot {M^s}} \right),} }$$

where $\odot$ is Hadamard product, $\varphi$: ${\mathbb{R}}{{}^{c \times h \times w}}$ $\mapsto$ ${\mathbb{R}}{{}^{hw \times c}}$ refers to the reshape function, and ${M^s}$ denotes the support mask.

Then, by measuring the cosine similarity of the inner product angle, the risk of overfitting of class-specific information brought by the feature specification is reduced, and the correlation map is calculated as *C* $\in$ ${\mathbb{R}}{{}^{{h^q}{w^q} \times {h^s}{w^s}}}$ as

(3) $$C = {{\left\langle {{W^q}\widehat {{F^q}},{W^k}\widehat {{F^s}}} \right\rangle } \over {\parallel {W^q}\widehat {{F^q}}\parallel \parallel {W^k}\widehat {{F^s}}\parallel t}},$$

where ${W^q}$ and ${W^k}$ $\in {\mathbb{R}}{{}^{c \times c}}$ represent the learnable parameters, $\parallel \cdot \parallel$ signifies the ${L^2}$ norm, and $t$ is a hyperparameter utilized to control the distribution range, which is empirically set to 0.1 across all experiments. HDMNet introduces the inverse softmax layer, which normalizes the correlation matrix along the query axis, adhering to the principle that

(4) $$\hat C\left( {i,j} \right) = {{\exp \left( {C\left( {i,j} \right)} \right)} \over {\sum\nolimits_{k = 1}^{h_l^qw_l^q} {\exp } \left( {C\left( {k,j} \right)} \right)}}.$$

This aspect is pivotal, as the objective is to retrieve only the region of interest within the query set. This ensures that the model concentrates on the relevant features during the segmentation task, thereby enhancing its precision and effectiveness.

Finally, the prior mask ${M^{pri}}$ $\in {\mathbb{R}}{{}^{{h^q} \times {w^q}}}$ is introduced and concatenated with the corresponding score along the channel dimension to generate a matching result

(5) $$A = {W^\circ }\left( {\left[ {\psi \left( {\hat C\left( {{W^v}\widehat {{F^s}}} \right)} \right),{M^{pri}}} \right]} \right),$$

where ${W^v} \in {\mathbb{R}}{{}^{c \times c}}$, ${W^\circ } \in {\mathbb{R}}{{}^{c \times \left( {c + 1} \right)}}$ denote the learnable parameters, $\widehat {{F^s}} \in {\mathbb{R}}{{}^{{h^s}{w^s} \times c}}$, $A \in {\mathbb{R}}{{}^{c \times {h^q} \times {w^q}}}$ are flattened support features and matching output, and $\psi :{\mathbb{R}}{{}^{{h^q}{w^q} \times c}} \;\mapsto \; {\mathbb{R}}{{}^{c \times {h^q}{ \times ^q}}}$ is the reshape function.This correlation mechanism computes pixel-level correspondence without directly relying on semantic-specific features, thereby alleviating the training set overfitting problem.

HDMNet introduces the concept of correlation map distillation, a technique that nudges the shallow layers to emulate the semantic relevance found in deeper layers, thereby enabling the former to comprehend the context more effectively and make high-quality predictions.

In Eq. (4), the correlation maps $\left\{ {{C_l} \in {\mathbb{R}}{{}^{h_l^qw_l^q \times h_l^sw_l^s}}} \right\}_{l = 1}^L$ for the query and support features are calculated. Subsequently, ${C_l}$ is reorganized through mean averaging, and irrelevant information is filtered out using the support mask ${M^s}$, as follows:

(6) $$C_l^\prime \left( i \right) = {{\mathop \sum \nolimits_{j = 1}^{h_l^sw_l^s} {C_l}\left( {i,j} \right) \cdot \left[ {\varphi \circ {\zeta _l}\left( {{M^s}} \right)\left( j \right) \; > \; 0} \right]} \over {\mathop \sum \nolimits_{j = 1}^{h_l^sw_l^s} \left[ {\varphi \circ {\zeta _l}\left( {{M^s}} \right)\left( j \right) \; > \; 0} \right]}},$$

where $l$ indicates the stage, $C_l^\prime \in {\mathbb{R}}{{}^{h_l^qw_l^q}}$, and ${\zeta _l}$ is the resize function. Given the flattened correlation maps, a softmax layer is applied to perform spatial normalization among all positions, resulting in the normalized maps denoted as:

(7) $$\widehat {C_l^\prime }\left( i \right) = {{\exp \left( {C_l^\prime \left( i \right)/T} \right)} \over {\mathop \sum \nolimits_{j = 1}^{h_l^qw_l^q} \exp \left( {C_l^\prime \left( j \right)/T} \right)}}$$

In this context, $l$ signifies the stage, and *T* represents the temperature of distillation (Hinton, Vinyals & Dean, 2015), which is set to 1.

Subsequently, the KL (Kullback-Leibler) divergence loss is employed to supervise from the teacher to the student, utilizing their softmax output. The correlation maps of adjacent stages serve as the teacher and student, respectively, and are formulated as follows:

(8) $$ \matrix{\displaystyle {{{{\mathscr{L}}}_{KL}} = \sum\nolimits_{a \in {    {\mathscr{A}}}     } {{\phi _t}} \left( a \right)\log \left( {{{{\phi _t}\left( a \right)} \over {{\phi _s}\left( a \right)}}} \right)}  \cr  \;\;\;\;\;\;\;{\displaystyle = \mathop \sum \nolimits_{i = 1}^{h_l^qw_l^q} {\zeta _l}\left( {{{\hat C}_{l + 1}}} \right)\left( i \right) \cdot \log \left( {{{{\zeta _l}\left( {{{\hat C}_{l + 1}}} \right)\left( i \right)} \over {{{\hat C}_l}\left( i \right)}}} \right),}  \cr  }$$

where $l$ indicates the stage, ${\phi _t}$ is the teacher model while ${\phi _s}$ is the student model, and ${\zeta _l}:{\mathbb{R}}{{}^{h_{l + 1}^qw_{l + 1}^q}} \mapsto {\mathbb{R}}{{}^{h_l^qw_l^q}}$ represents resizing. Specifically, for the final correlation map that lacks a successor, the ground truth is directly employed as its teacher.

### Sharpness-aware minimization

Applying few-shot segmentation to poultry farming poses significant challenges due to the high variability in poultry appearance and behavior, alongside the complex and dynamic backgrounds of farm environments. These factors contribute to overfitting in conventional few-shot learning models, wherein the model becomes excessively tailored to the limited training data and struggles to generalize effectively to new, unseen data.

To mitigate this issue, we have integrated sharpness-aware minimization (SAM) into the HSDNet model’s learning process. SAM, an advanced optimization technique, targets the smoothness of the loss landscape, which is crucial for managing overfitting. By strategically minimizing the sharp minima within the loss function, SAM ensures that the model does not overly fit the specificities of the limited training data, thereby enhancing its generalization capabilities. This approach is particularly advantageous in the context of poultry farming, where the diversity in visual data—owing to variations in breeds, sizes, and behaviors of poultry, as well as fluctuating farm conditions—demands robust modeling techniques.

**Figure 3:**
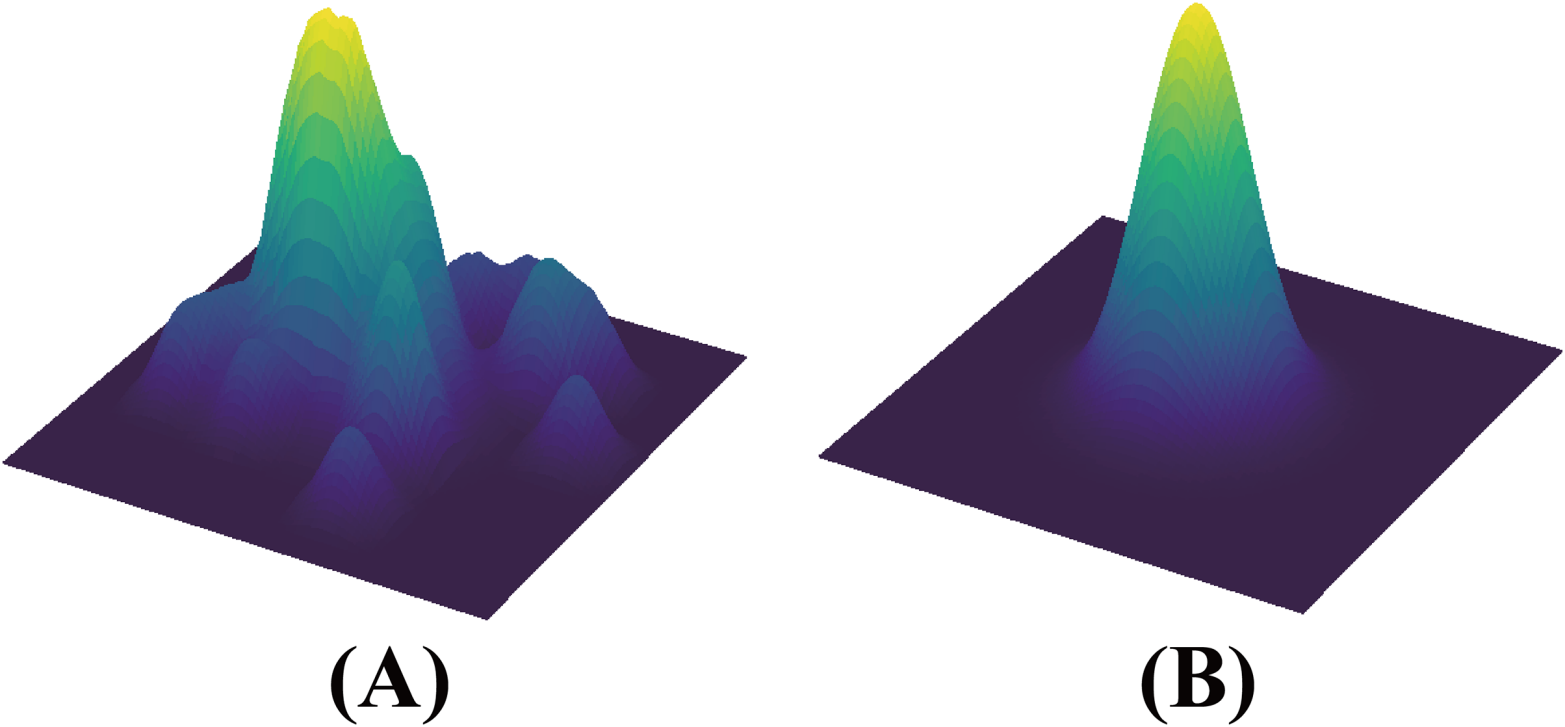
(left) A sharp minimum to which a ResNet trained with SGD converged. (right) A wide minimum to which the same ResNet trained with SAM converged. Data sourced from the SAM.

The introduction of SAM enables the HSDNet model to develop robust, transferable features, significantly improving its generalization across different environments. As illustrated in Fig. 3, the comparison of loss landscapes between networks with and without residual connections demonstrates this effect. In the absence of these connections, the HSDNet’s loss landscape appears rugged—characterized by steep inclines, sharp peaks, and profound valleys. Conversely, with the inclusion of residual connections, the landscape becomes markedly smoother, leading the HSDNet model to flatter convergence points or local minima. This smoothness is critical for the model’s performance in accurately segmenting new, unseen poultry images that substantially deviate from the training set. Through the incorporation of SAM, the HSDNet model adapts more effectively to novel environments and variations in poultry, thereby boosting the efficacy of few-shot segmentation models in agricultural applications.

To enhance the HSDNet model, understanding the complexities of model optimization in deep learning is crucial. Modern deep learning models, often over-parameterized, face a challenge: training loss may not reliably indicate generalization capability. Relying solely on this metric can result in sub-optimal performance. Addressing this, Pierre Foret and colleagues, inspired by research on the geometric characteristics of loss landscapes and their impact on generalization, introduced Sharpness-Aware Minimization (SAM) (Foret et al., 2020). SAM focuses on reducing loss while ensuring the smoothness of the loss curve. Unlike traditional optimization methods that may converge to sharp minima and cause instability, SAM aims for a smoother minimum, enhancing the model’s stability and generalization. This approach is formally presented as follows:

(9) $$ \mathop {\min}_{\theta} \limits \; \max_{||\epsilon|| \leq \rho}\; {{\mathscr{L}}}_{obj}(\theta + \epsilon).$$

The function ${{{\mathscr{L}}}_{obj}}$ stands as a pivotal entity, representing the objective function in deep learning optimization. This function plays a crucial role by quantifying the divergence between the predicted and actual outcomes, with the overarching aim of the HSDNet model being to minimize this discrepancy throughout the training process.

The term $\rho \ge 0$, a predetermined hyperparameter, delineates the upper boundary for the magnitude of $\epsilon$, ensuring that its norm does not surpass $\rho \ge 0$.

Navigating through this optimization landscape, a notable challenge emerges when attempting to pinpoint the exact solution for the inner maximization, attributed to the inherent complexity of the problem. SAM, in response to this, employs a first-order approximation technique. This approach simplifies the problem by leveraging only the first derivative of the function, as follows:

(10) $$\eqalign{ \hat{\epsilon}(\theta) \approx &  \mathop {\arg \max}_{||\epsilon|| \leq \rho} \limits \; L_{obj}(\theta) + \epsilon^{\mathbf{T}}\nabla_{\theta} L_{obj}(\theta) \cr =  &  \rho \nabla_{\theta} L_{obj}(\theta) / ||\nabla_{\theta} L_{obj}(\theta)||_2 .}$$

During the optimization process, the modified term $\hat {\epsilon }(\theta )$ is amalgamated with the weight parameters $\theta$ to formulate a new, adjusted weight. This amalgamation ensures a more efficacious adjustment in the direction where the objective function, ${{{\mathscr{L}}}_{obj}}$, can be potentially minimized.

Gradients, in this context, serve as a linchpin in updating the model. Specifically, the gradient adjustment for the weights $\theta$ is computed using $\nabla _{\theta } \mathcal {L}_{obj}(\theta )|_{\theta + \hat {\epsilon }(\theta )}$. This particular formulation considers the impact of the aforementioned modified term on the gradient, enabling a more nuanced update strategy. The aforementioned procedure can be perceived as a generic formulation that enhances smoothness for any ${{{\mathscr{L}}}_{obj}}$.

This entire mechanism is not merely a serendipitous construct but a meticulously devised approach aimed at enhancing the smoothness of the HSDNet model’s learning curve. The objective is to render the optimization landscape smoother and, consequently, more navigable, which is pivotal for achieving optimal convergence.

To elaborate, HSDNet introduces a concept termed the ‘sharpness-aware source risk.’ This concept is designed to refine the process, focusing on identifying regions in the optimization landscape that are not only low in value (indicating minima) but also demonstrate a desirable level of smoothness. Accordingly, HSDNet employs the sharpness-aware source risk for the identification of a smooth minimum:

(11) $$\mathop{\max}_{||\epsilon|| \leq \rho}\limits \; {R}_{S}^l(h_{\theta + \epsilon}) = \mathop{\max}_{||\epsilon|| \leq \rho} \limits {\mathbb{E}}_{x \sim P_S}[\; l(h_{\theta + \epsilon}(x), f(x))],$$

SAM also now defines the sharpness aware discrepancy estimation objective below:

(12) $$\max_{\Phi} \min_{||\epsilon|| \leq \rho} d_{S}^{\Phi + \epsilon}.$$

As the sharpness-aware objective aims to maximize $d_S^\Phi$, it employs $\mathop {\min }_{||\epsilon || \leq \rho } \limits$ rather than $\mathop {\max }_{||\epsilon || \leq \rho } \limits$ to seek smoother maxima. The discrepancy estimation difference between the smooth version $d_S^{{\Phi ^{\prime \prime }}}$ (Eq. 12) and the non-smooth version $d_S^{{\Phi ^\prime }}$ is then theoretically analyzed. The theorem states that, assuming ${{\mathscr{D}}}\Phi$ is an *L*-smooth function (a common assumption for non-convex optimization), $\eta$ is a small constant, and $d{S^*}$ is the optimal discrepancy. The complete pseudocode for the SAM algorithm, employing SGD as the base optimizer, is presented as Algorithm 1.

**Algorithm 1:** SAM algorithm.

1: **Input**: Training set ${{\mathscr{J}}}{\mathrm{ \mathop =^\Delta \limits }} \cup _{i = 1}^n\{ ({{{x}}_i},{{{y}}_i})\}$, Loss function $l:{{\mathscr{W}}} \times {{\mathscr{X}}} \times {{\mathscr{Y}}} \to {\mathbb R{_ + }}$, Batch size *b*, Step size $\eta \; > \; 0$, Neighborhood size $\rho \; > \; 0$.
2: **Output**: Model trained with SAM
3: Initialize weights *w*_0_, $t = 0$;
4: **while** *notconverged* **do**
5: Sample batch ${\cal B} = \{ ({x_1},{y_1}), \ldots ({x_b},{y_b})\} $;
6: Compute gradient ${\nabla _w}{L_{\cal B}}(w)$ of the batch's training loss;
7: Compute $\hat{\boldsymbol{\epsilon }}(\boldsymbol{w})$ per Eq. (15);
8: Compute gradient approximation for the SAM objective (Eq. (17)): ${\boldsymbol g} = \nabla_w L_{{\cal B}}({\boldsymbol w})\mid _{{\boldsymbol w} + \hat{\boldsymbol {\epsilon }}({\boldsymbol w}) }$;
9: Update weights: ${{{w}}_{t + 1}} = {{{w}}_t} - \eta {{g}}$;
10: $t = t + 1$;
11: **end while**
12: **return** ${{\bf{w}}_t}$

### Dice loss

Within the field of poultry farming, a prevalent issue is the disparity between the quantities of positive and negative samples. This imbalance arises because target areas in poultry images, such as specific parts of the chickens, often constitute only a small portion of the overall image. Traditional pixel-level loss functions, like cross-entropy, may struggle to effectively manage this imbalance. Consequently, models tend to bias towards larger background areas, neglecting smaller yet critical target regions, leading to difficulties in model convergence. The issue is further exacerbated in few-shot segmentation scenarios. Poultry images can exhibit high variability, such as differing postures, sizes, or environmental backgrounds, and the limited availability of annotated samples. This scenario can hinder segmentation models from capturing all essential features during the learning process, increasing the risk of model overfitting.

HSDNet incorporates Dice loss, a loss function particularly effective for segmentation tasks. Unlike traditional loss functions, Dice loss quantifies the performance of the segmentation model by measuring the overlap between the predicted segmentation and the ground truth, providing a more direct assessment of segmentation quality. This loss function is inherently designed to handle imbalances between positive and negative samples by evaluating both correctly and incorrectly predicted areas simultaneously. Such an approach is beneficial for segmenting poultry from complex backgrounds as it enhances the model’s ability to discern subtle differences, thereby improving its generalization capabilities across varied environmental conditions.

The theoretical foundation for choosing Dice loss lies in its derivation from the Dice coefficient, a statistical tool named after its creator, which is used to gauge the similarity between two sets. A higher Dice coefficient, indicating greater similarity, translates directly to improved accuracy in the context of segmentation tasks. This relevance makes Dice loss an optimal choice for the HSDNet model, enhancing its robustness and performance in challenging segmentation scenarios. The mathematical representation of the Dice coefficient is as follows:

(13) $$Dice = {{2|X \cap Y|} \over {|X| + |Y|}},$$

where $|X \cap Y|$indicates the number of intersections between X and Y, and |*X*| and |*Y* | do not indicate the number of elements in X and Y. The Dice loss expression is as follows:

(14) $$DiceLoss = 1 - Dice = 1 - {{2|X \cap Y|} \over {|X| + |Y|}},$$

When Dice loss is often used in semantic segmentation problems, X represents the pixel label of the real segmentation image, Y represents the pixel category of the model prediction segmentation image, $|X \cap Y|$ is approximately the dot product between the pixel of the predicted image and the pixel of the real label image, and the dot product result is added. |*X*| and |*Y* | are approximately added as pixels in their respective images, respectively.

A pivotal advantage of Dice loss is its capability to counteract imbalances between foreground and background. In scenarios where the foreground area is minimal and the background is predominant, this imbalance can skew training outcomes in numerous models. Specifically, the model may prioritize optimizing the prediction of the expansive background area due to its substantial contribution to the overall loss, thereby overlooking the smaller foreground areas. Dice loss enhances foreground recognition, ensuring a reduced false negative rate (FN). However, it also presents a challenge known as loss saturation, where it may cease to provide meaningful gradients as the model’s predictions increasingly align with the actual labels.

Conversely, the original loss function directly computes a loss for each pixel, correlating with the discrepancy between the predicted and actual label values of that pixel. Thus, the original loss values every pixel’s prediction equally, irrespective of whether it is part of the foreground or background. Nonetheless, this egalitarian approach can be problematic as the model might over-optimize the abundant background pixels, neglecting the foreground regions.

Given these considerations, exclusive reliance on Dice loss often falls short of yielding optimal segmentation results. To circumvent the limitations inherent to a singular loss function, the HSDNet model amalgamated both loss functions. Thus, the HSDNet model did not entirely forsake the original loss function but integrated Dice loss into it. Specifically, the final loss function is articulated as:

(15) $$Los{s_{new}} = \alpha Los{s_{orig}} + \beta Los{s_{Dice}},$$

where $\alpha$ and $\beta$ are weight coefficients that can be determined through cross-validation for optimal performance.

## Dataset

To rigorously evaluate the effectiveness of few-shot semantic segmentation in poultry farming, the study concentrated on common poultry species: geese, ducks and chickens. In addition to the aforementioned aspects, to demonstrate the robustness and generalizability of the HSDNet model, we have incorporated an aquatic species, specifically the fish (golden crucian carp), into our study. This inclusion serves to substantiate our assertion that the HSDNet model is not confined to poultry farming but has the potential for extensive application across a broad spectrum of agricultural domains. We gathered data from various rearing practices, including free-range and caged settings. Through our efforts in collecting and organizing previous studies, we have formulated the first few-shot poultry farming dataset encompassing multiple species. This dataset not only serves the purposes of our study but also offers a valuable resource for future research in this domain. Initially, the duck, goose, and fish datasets were derived from previous studies, all of which were affiliated with the Big Data Application Laboratory of Sichuan Agricultural University, to which the present study is also affiliated, and thus have research access rights.

### Dataset collection

**Figure 4:**
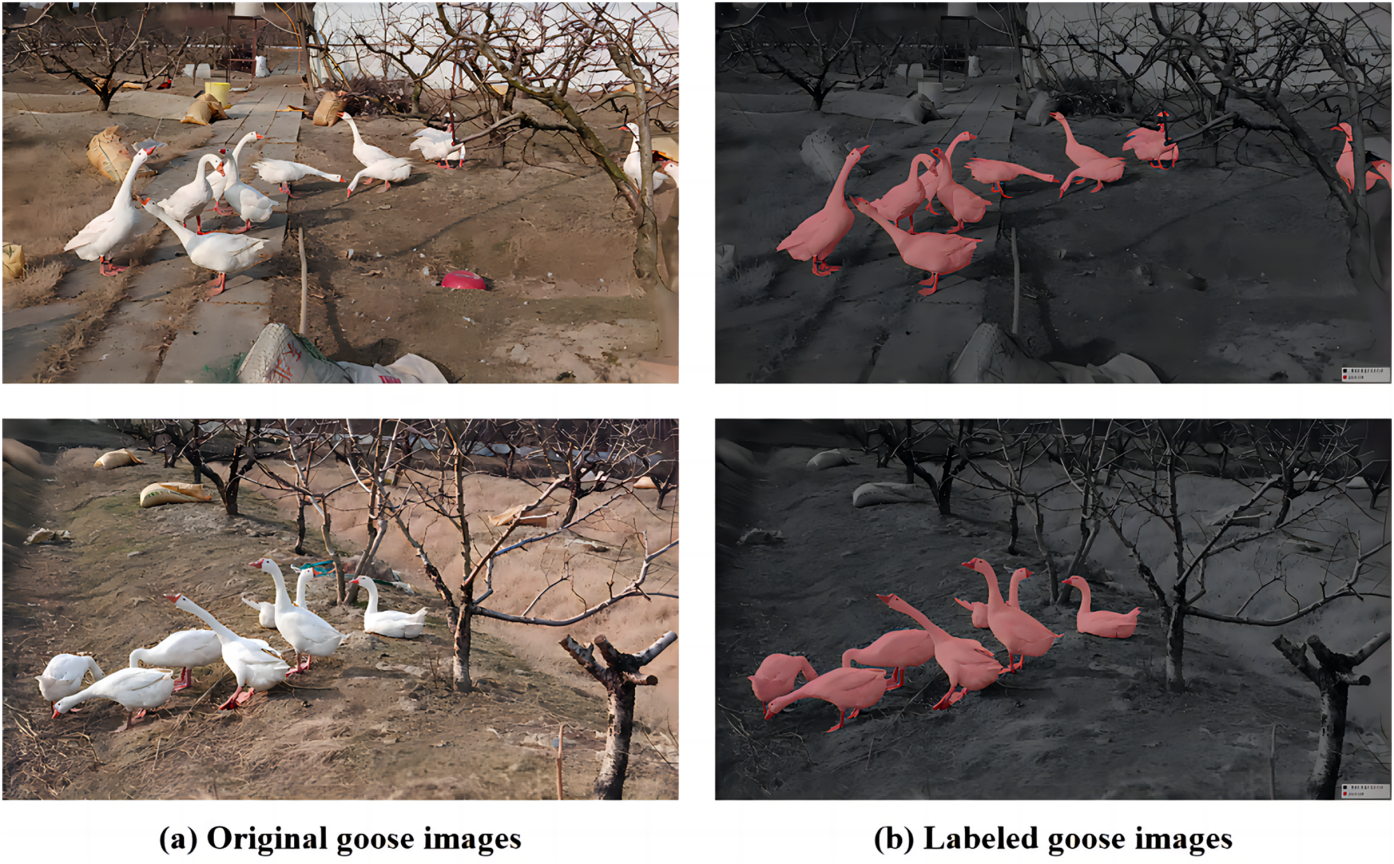
Goose breeding data set and labeling diagram. (A) The original image represents the original dataset image. (B) Annotate a schematic representation of data set labels. Figure source credit: https://github.com/DaixianLiu/DaixianLiu.github.io/tree/main/goose.

As for the goose dataset, it was provided by a private goose farm in Zhejiang Province, China. This goose dataset was meticulously annotated by members of our project team. Detailed information and resources for the goose dataset can be found at the following link: goose. This farm employs a free-range rearing method, providing the geese with a more natural growing environment, resulting in superior meat quality. Data was captured using the DJI Pocket 2 device device. After processing, the result was 639 images of acceptable quality, and the dimensions were set at 1,920 $\times$ 1,080 for annotation purposes. We drew from experience in annotating data using the labelme tool and the PASCAL VOC dataset format. A subset of the final dataset, including images and annotations, is illustrated in Fig. 4. The free-range setting presents a diverse and complex environment with various vegetation, flowing water, and other shading factors. This introduces significant noise and challenges to the dataset, making the experiments more demanding. The physical appearances of individual geese are highly similar, making it difficult, even for the human eye and neural networks, to distinguish specific geese. This poses challenges for later group analysis, emphasizing the importance of enhancing segmentation accuracy.

**Figure 5:**
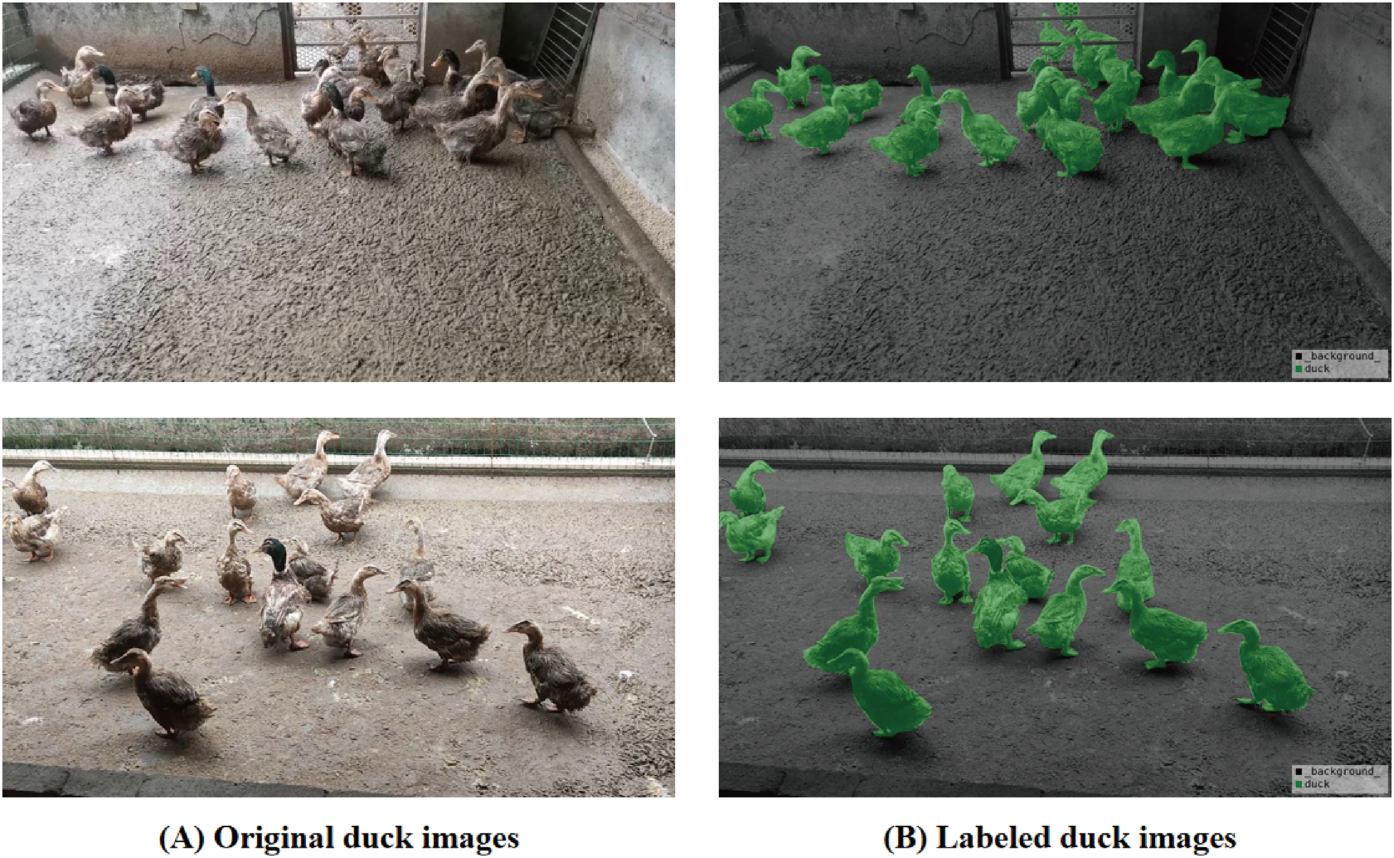
Duck breeding data set and labeling diagram. (A) The original image represents the original dataset image. (B) Annotate a schematic representation of data set labels. Figure source credit: https://github.com/DaixianLiu/DaixianLiu.github.io/tree/main/duck.

The duck dataset originates from the original waterfowl farm in Ya’an, Sichuan Province, China, focusing on the characteristic Ya’an hemp duck breed. The duck dataset, annotated by our project members, is available at the link duck. This farm employs a standardized caged-rearing method. While the caged environment is simpler compared to free-range, it has a higher breeding density. The data was captured using the DJI Pocket 2 device. After processing, a final dataset of 1,500 images of acceptable quality was obtained, with image dimensions standardized at 960 $\times$ 540 for annotation purposes. Drawing from prior experience, the data was annotated using the labelme tool and the PASCAL VOC dataset format. A portion of the finalized dataset, including images and annotations, is depicted in Fig 5. Given the high density of standardized caged rearing, the HSDNet model aims to address the accuracy issues associated with densely packed objects, particularly at the edges.

**Figure 6:**
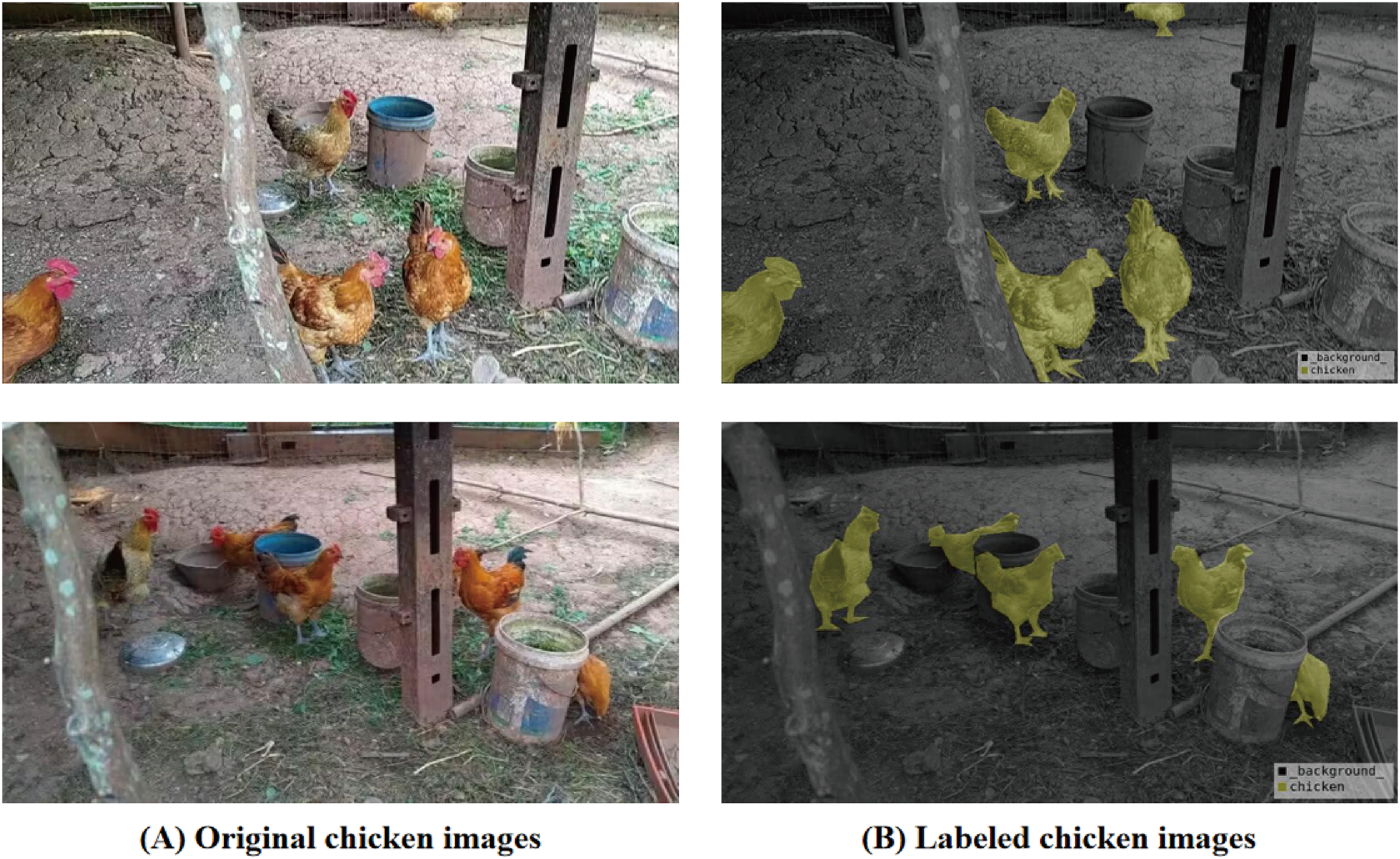
Chicken breeding data set and labeling diagram. (A) The original image represents the original dataset image. (B) Annotate a schematic representation of data set labels. Figure source credit: https://github.com/DaixianLiu/DaixianLiu.github.io/tree/main/chicken.

The chicken dataset is derived from several rural family farms in Ya’an, Sichuan Province, China, focusing on the laying hens breed commonly found in the Sichuan region. Our team members obtained this through on-site visits and collections. The dataset link for chicken is chicken. The recording was done using the smartphone, featuring a pixel resolution of 1,280 $\times$ 720. Subsequently, we conducted random sampling at intervals of 100 frames, ultimately acquiring a dataset comprising 600 images. The data annotation was performed by our colleagues using the labelme tool and formatted according to the PASCAL VOC dataset standards. The environment and characteristics of these settings lie between free-range and large-scale caged rearing. Furthermore, due to the fact that these are family-owned farms, there may be substantial differences in the rearing environments across different households, which demands a high level of robustness from the HSDNet model. A subset of the finalized dataset, inclusive of images and annotations, is depicted in Fig. 6.

**Figure 7:**
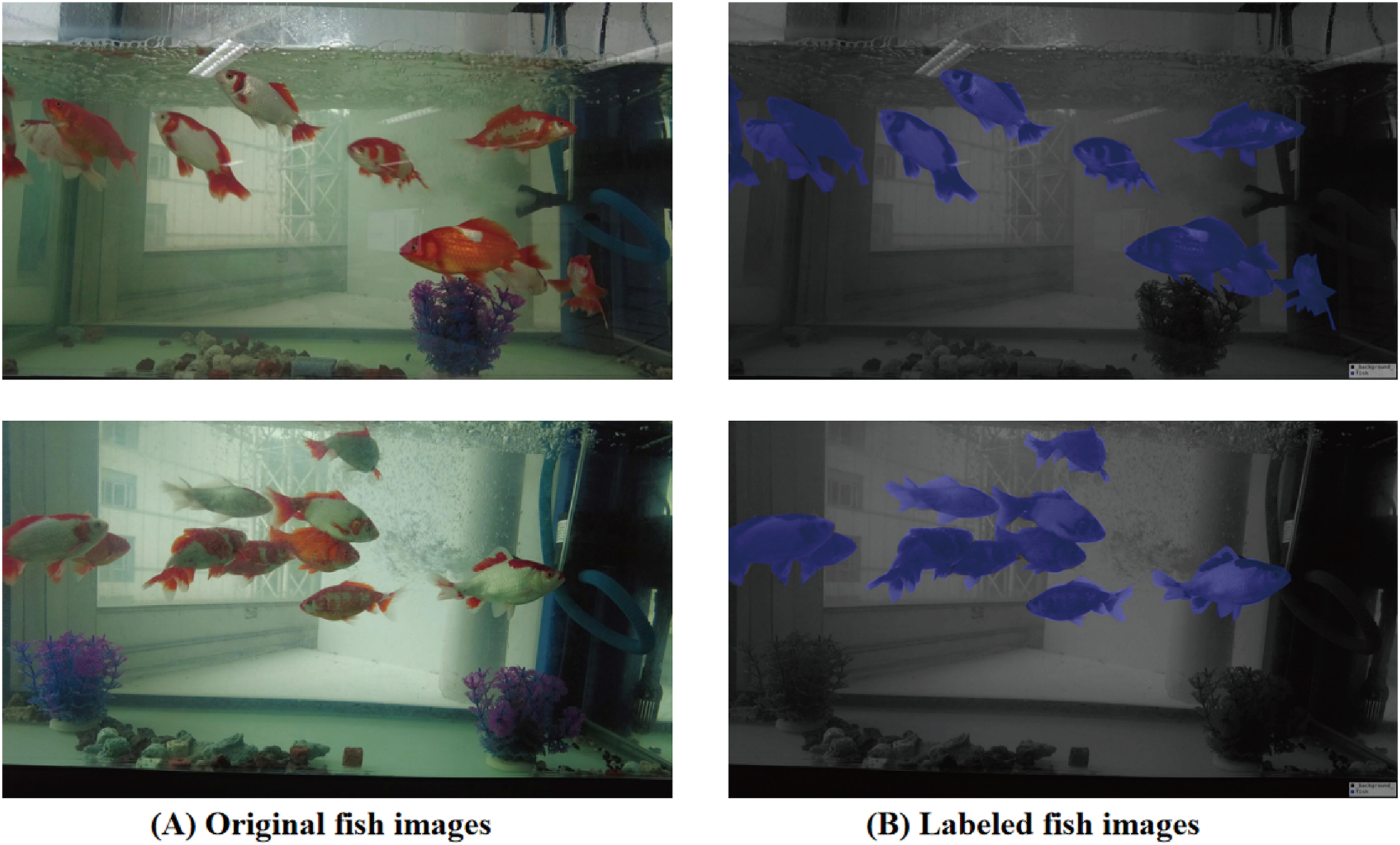
Fish breeding data set and labeling diagram. (A) The original image represents the original dataset image. (B) Annotate a schematic representation of data set labels. Figure source credit: https://github.com/DaixianLiu/DaixianLiu.github.io/tree/main/fish.

To further substantiate the robustness of the HSDNet model and facilitate its applicability to other domains, this study has integrated a dataset originating from an aquatic. The dataset link for fish is fish. The fish, sourced from a transparent ornamental tank and annotated by our team members, had the golden crucian carp as the focal point of our research. The data was captured using the DJI Pocket 2, boasting a resolution of 1,920 $\times$ 1,080. By sampling the video at 30 frames per second, a total of 500 high-quality images were amassed. A subset of this dataset, alongside annotations, is showcased in Fig. 7. The underwater environment, with its distinct lighting conditions, buoyancy factors, and dynamic movements, presents unique challenges to the HSDNet model. Through juxtaposing terrestrial and aquatic organisms in comparative experiments, the study demonstrates that the HSDNet model effectively overcomes limitations in model generalization caused by varying species.

## Experiments

### Experiments setting

The study conducted a comprehensive evaluation of the HSDNet model’s performance on the poultry farming dataset. Geese were used as the base class, with laying hens, hemp ducks, and golden crucian carp as new classes, meanwhile, fish were employed to validate the model’s extensibility. For the geese class, we obtained 1,391 high-quality images through methods such as data augmentation. For the laying hens, hemp ducks, and golden crucian carp classes, we each selected 60 images captured from various angles and at different times to ensure diversity and comprehensiveness in the dataset. The images in the dataset were resized to 473 $\times$ 473 to ensure that the HSDNet model runs efficiently.

We opted for the PyTorch framework to build, optimize, and evaluate the HSDNet model. All models were trained and tested in an environment equipped with two V100-32 GB GPUs, an Intel(R) Xeon(R) Gold 6130 CPU clocked at 2.10 GHz, PyTorch version 1.7.0, Python version 3.8, Cuda version 11.0, and running on the Ubuntu 18.04 operating system. Table 1 details the software and hardware setups used throughout the training and testing phases of our research.

**Table 1:** Software and hardware configurations.

Software	Type/version	Hardware	Type/version
PyTorch	1.7.0	GPUs	2 $\times$ NVIDIA V100-32 GB
Python	3.8	CPU	Intel(R) Xeon(R) Gold 6130 @ 2.10 GHz
CUDA	11.0	RAM	25 GB
OS	Ubuntu 18.04	Hard disk	80 GB

To quantify the performance of the HSDNet model, the study employed the mean intersection over union(mIoU) as our primary evaluation metric. mIoU is a commonly used metric in semantic segmentation tasks, measuring the overlap between the predicted segmentation regions and the ground truth annotations. The formula for mIoU is:

(16) $$mIOU = {1 \over N}\mathop \sum \nolimits_{i = 1}^N {{\mid {P_i} \cap {G_i}\mid } \over {\mid {P_i} \cup {G_i}\mid }},$$

where *N* is the total number of classes, ${P_i}$ represents the region of the ith class predicted by the model, ${G_i}$ represents the region of the ith class in the ground truth, ${P_i} \cap {G_i}$ denotes the size of the intersection between the predicted and ground truth regions, ${P_i} \cup {G_i}$ denotes the size of the union between the predicted and ground truth regions. Through mIoU, we can gain a more intuitive understanding of the model’s segmentation performance across various classes, thus evaluating its overall efficacy.

We utilized ResNet-50 (He et al., 2016) as our encoder to extract features with frozen parameters. PSPNet (Zhao et al., 2017) served as the foundational learner for all experiments. The Pyramid Pooling Module (PPM) (Zhao et al., 2017) was employed after the 4th block of ResNet-50 to provide a multi-resolution context for enriched features, facilitating the generation of a prior mask. We trained in an ensemble manner on the poultry farming dataset with a batch size set to 8. During training, we adopted the AdmW optimizer with a learning rate of 0.0001 and a weight decay of 0.01. At the same time, due to the fast convergence of AdamW, we set the epoch to 100 to guarantee effective convergence. Additionally, we adjusted the learning rate using the “poly” strategy.

### Comparative experiments

In the quest to select a model suitable for few-shot semantic segmentation of poultry, the study trained and tested the poultry farming dataset on prevalent few-shot semantic segmentation models (BAM Lang et al., 2022, MIANet Yang et al., 2023, HDMNet Peng et al., 2023) and compared their miou performance metrics. Given the superior performance of HDMNet on the poultry farming dataset compared to the other models, we opted for HDMNet as our primary model for few-shot semantic segmentation, further refining and conducting subsequent experiments based on it. The detailed experimental results are presented in Table 2.

**Table 2:** 1-shot few-shot segmentation experiment.

Methods		Chicken	Duck	Fish	Avg
Traditional segmentation	PSPNet	25.39	25.88	27.99	26.42
	UNet	44.80	41.62	40.28	42.23
	DeepLabv3+	46.76	43.42	43.06	44.41
	SeaFormer	38.41	38.29	39.35	38.68
Few-shot segmentation	BAM	80.15	59.70	65.82	68.56
	MIANet	58.25	**79.44**	67.62	68.44
	HDMNet	77.64	61.12	67.80	68.85
	HSDNet(Ours)	**81.37**	67.77	**69.54**	**72.89**

**Note:**

The best results are shown in bold.

As demonstrated in Table 2, the HSDNet model significantly outperforms other few-shot semantic segmentation models on the poultry farming dataset across comprehensive metrics. Specifically, in a 1-shot scenario, the HSDNet model’s miou is 6% higher than that of BAM and MIANet, and 5.8% higher compared to the baseline model, HDMNet. This achievement marks HSDNet as the top performer in the miou metric. Notably, in the 1-shot setting, the iou for chicken images stands at 81.37%, while for duck images it’s 67.77%. Moreover, when applied to aquatic species, HSDNet demonstrates robust versatility with an iou of 69.54% for fish images. Similarly, as shown in Table 3, for 5-shot, the HSDNet model achieves the highest miou, successfully meeting our research objectives. These findings solidify HSDNet’s leading position in small-scale intensive poultry environments and highlight its potential for broader agricultural applications.

**Table 3:** 5-shot few-shot segmentation experiment.

Methods		Chicken	Duck	Fish	Avg
Traditional segmentation	PSPNet	61.92	60.19	52.88	58.33
	UNet	70.94	63.94	67.95	67.61
	DeepLabv3+	72.87	57.45	55.68	62.00
	SeaFormer	68.33	56.28	60.12	61.58
Few-shot segmentation	BAM	80.42	63.57	72.40	72.13
	MIANet	58.25	**79.42**	66.22	67.96
	HDMNet	79.58	68.65	70.89	73.04
	HSDNet(Ours)	**80.98**	65.96	**75.81**	**74.25**

**Note:**

The best results are shown in bold.

In addition to the aforementioned results, we have further showcased the superiority of our model over traditional semantic segmentation models. As illustrated in Tables 2 and 3, regardless of whether it’s the 1-shot or 5-shot scenario, the HSDNet model’s miou significantly outperforms that of conventional semantic segmentation models. Given our choice of PSPNet as the foundational learner for the HSDNet model, a comparison with recent traditional semantic segmentation models, as presented in Tables 2 and 3, reveals that their miou scores surpass that of PSPNet. This indicates that by changing the base learner, there’s potential to further elevate the HSDNet model’s performance. Nevertheless, this experiment has already substantiated the efficacy of the HSDNet model.

**Figure 8:**
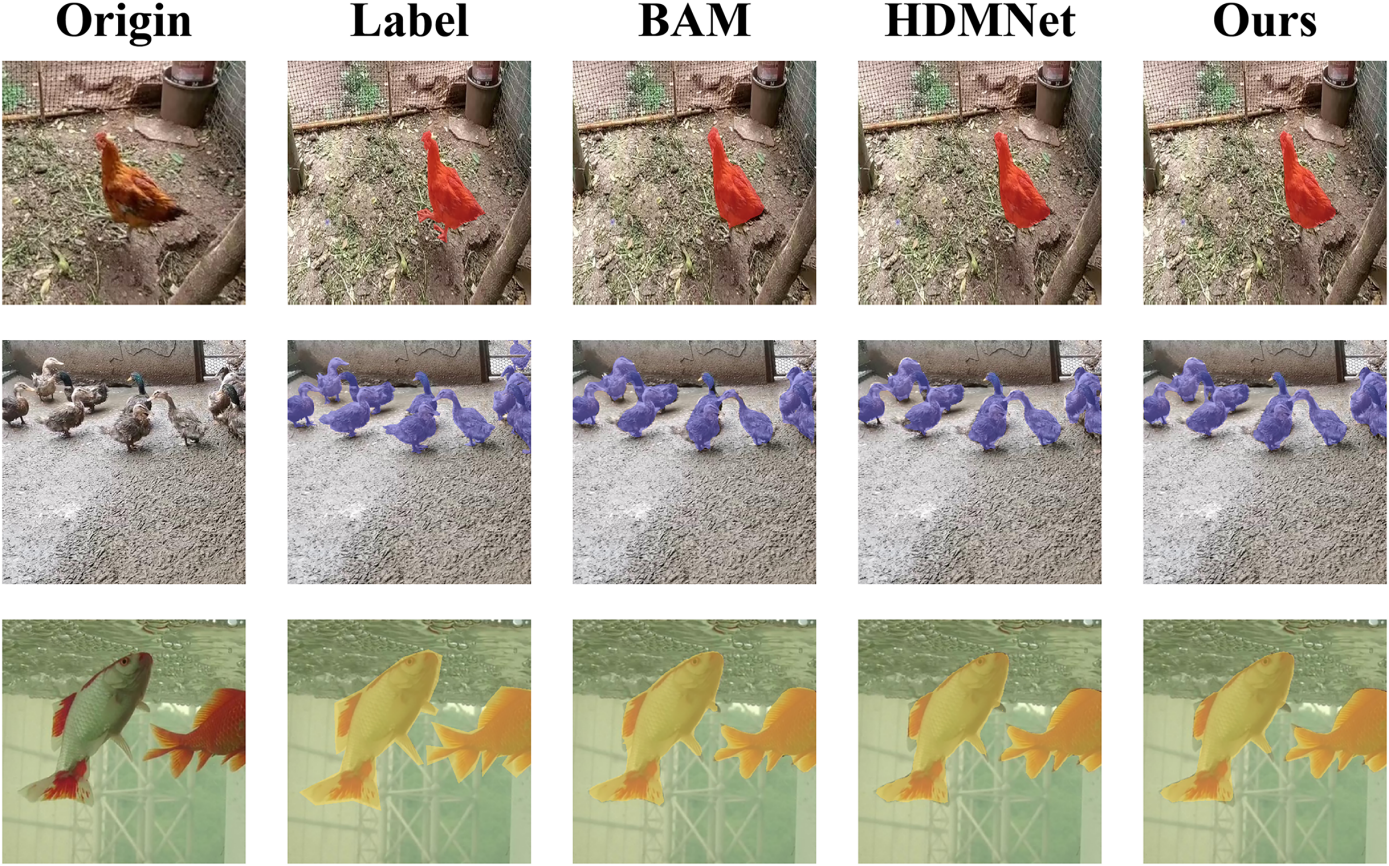
Visualization of 1-shot results. The first column displays the original images, and the second column presents the corresponding annotated images, expressing the ideal segmentation outcomes we aspire for the model to learn and predict. The following three columns individually showcase the effect images from various models in the semantic segmentation task. Figure source credits: https://github.com/DaixianLiu/DaixianLiu.github.io/tree/main/chicken, https://github.com/DaixianLiu/DaixianLiu.github.io/tree/main/duck, https://github.com/DaixianLiu/DaixianLiu.github.io/tree/main/fish.

Figure 8 visually demonstrates HSDNet’s exceptional capabilities using a 1-shot example. When compared to the BAM model, specifically looking at the chicken in Fig. 8, it is apparent that the BAM model’s segmentation output fails to adequately cover the chicken’s tail. In contrast, our HSDNet model demonstrates its exceptional ability to capture the finest details of the chicken, including the tail, ensuring a comprehensive and precise mask. Similarly, when compared with HDMNet, particularly with respect to the duck shown in Fig. 8, it is clear that HDMNet struggles to fully mask the duck. This shortcoming is most evident on the left side of the image it produces. Meanwhile, our HSDNet model effectively masks the duck, highlighting HSDNet’s practical superiority in achieving high-fidelity semantic segmentation.

**Figure 9:**
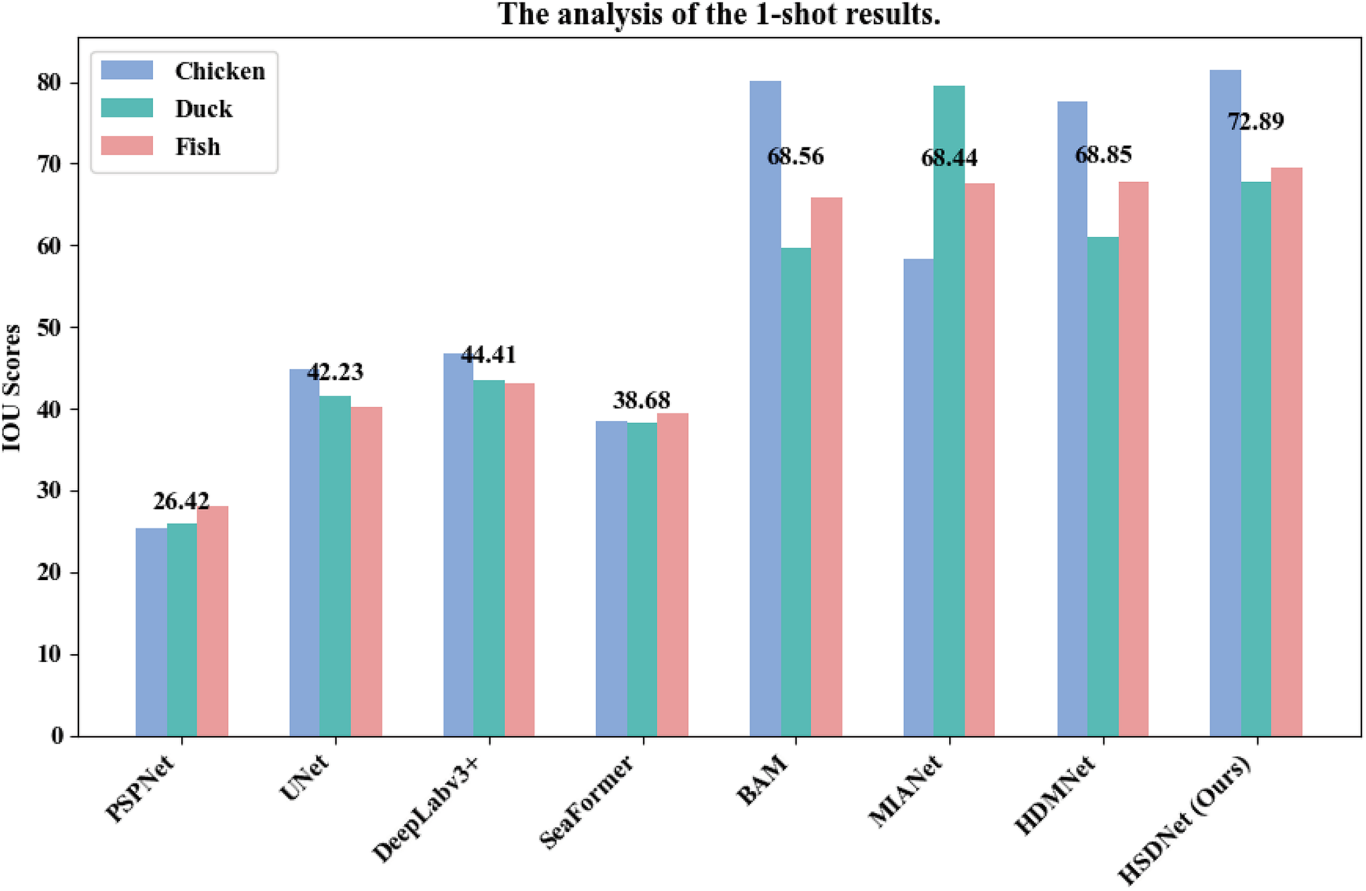
1-shot grouped bar chart. The horizontal axis lists evaluated models, from traditional to few-shot segmentation methods. The vertical axis shows the IOU scores, measuring segmentation accuracy. Data sourced from the 1-shot bar.

**Figure 10:**
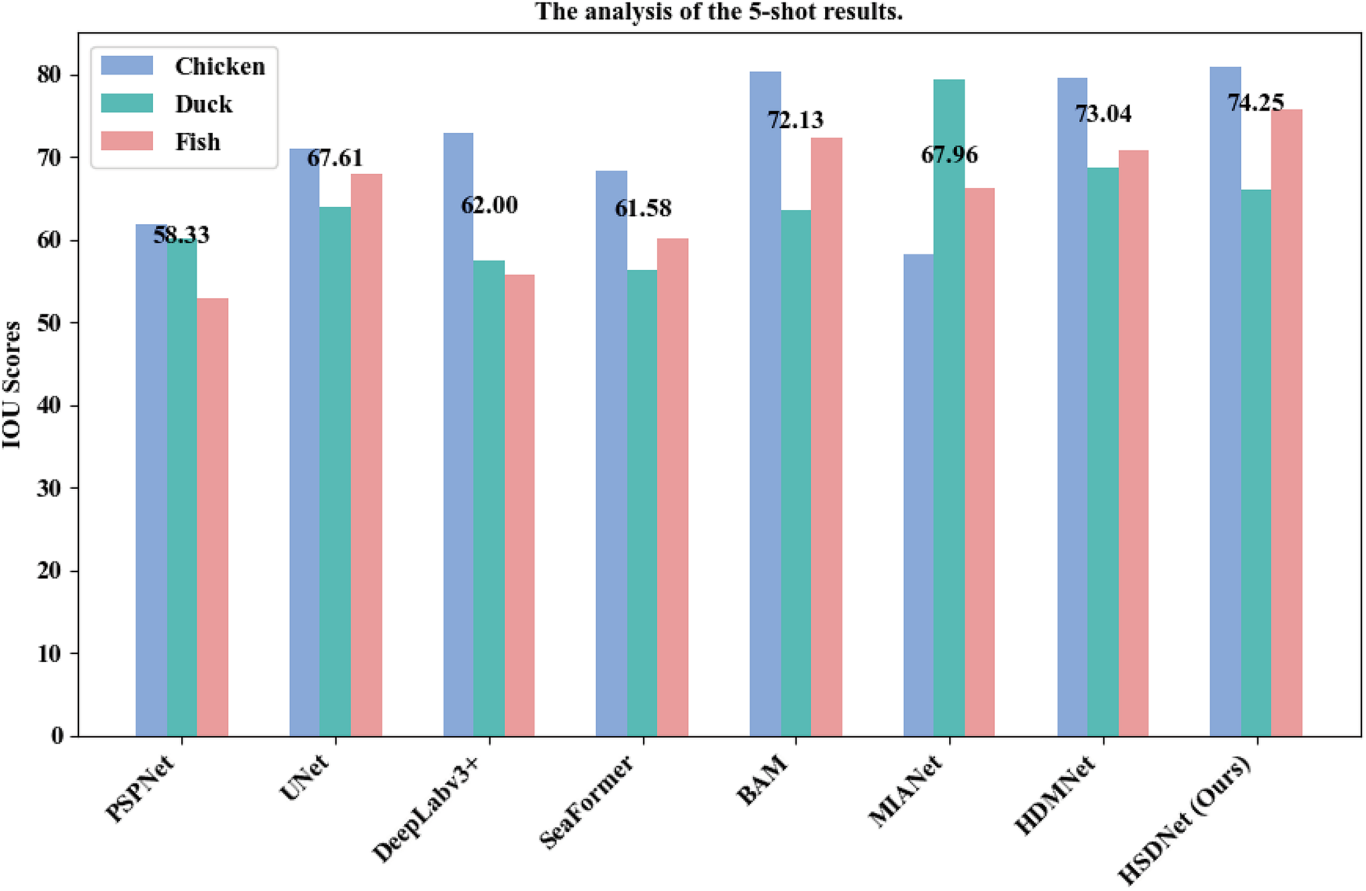
5-shot grouped bar chart. The horizontal axis lists evaluated models, from traditional to few-shot segmentation methods. The vertical axis shows the IOU scores, measuring segmentation accuracy. Data sourced from the 5-shot bar.

Additionally, we have illustrated detailed charts for the 1-shot and 5-shot segmentation experiments in Figs. 9 and 10, respectively, which juxtapose the performance across three categories, namely Chicken, Duck, and Fish, showcasing a comparative analysis between our model, HSDNet, and other state-of-the-art approaches. These charts distinctly reveal that HSDNet boasts competitive IOU scores across different categories in the 1-shot setting and witnesses further enhancements in the 5-shot experiments, underscoring its superior adaptability and performance. Notably, in the Fish category, the bar corresponding to HSDNet is significantly higher, indicating its pronounced superiority over other methods and highlighting its efficiency in tackling more challenging segmentation tasks. The comparison of average IOU scores demonstrates that HSDNet consistently outperforms other methodologies across all tested categories, emphasizing its stability in handling a variety of segmentation tasks and affirming its robustness as a leading segmentation approach. These experimental outcomes vividly underline HSDNet’s significant advantages over existing state-of-the-art methods.

### Ablation experiment

We conducted a series of ablation studies to investigate the impact of each component on segmentation performance. It’s worth noting that unless specified otherwise, all ablation experiments were carried out on the poultry farming dataset under the 1-shot setting, utilizing the ResNet-50 (He et al., 2016) backbone.

We conducted four sets of ablation experiments: directly using the HDMNet model, introducing the Sharpness-Aware Model (Foret et al., 2020) based on HDMNet, adding diceloss to the HDMNet model, and simultaneously incorporating both the Sharpness-Aware Model and Dice loss to the HDMNet. The results of the ablation studies are illustrated in Table 4.

**Table 4:** Model ablation experiment.

model	SAM	Dice loss	Chicken	Duck	Fish	Avg
Baseline	×	×	77.64	61.12	67.80	68.85
	✓	×	79.87	65.00	68.53	71.13
	×	✓	79.04	64.28	67.70	70.34
HSDNet (Ours)	✓	✓	**81.37**	**67.77**	**69.54**	**72.89**

From Table 4, it is evident that introducing the Sharpness-Aware Model based on HDMNet results in a 3% improvement in mIoU compared to using HDMNet alone, demonstrating the effectiveness of incorporating the Sharpness-Aware Model into HDMNet. Adding diceloss to HDMNet leads to a 2% increase in mIoU compared to using HDMNet directly, indicating that the model’s performance is further enhanced with diceloss. Lastly, when both the Sharpness-Aware Model and diceloss are introduced to HDMNet, the results, as shown in the fourth row of Table 4, surpass the mIoU of the previous three sets, suggesting that the combined introduction of both components significantly boosts the model’s performance, achieving optimal and satisfactory results.

### Robustness analysis

To better demonstrate the robustness and scalability of our model, we conducted additional robustness experiments using the benchmark few-shot segmentation dataset PASCAL $- {5^i}$ (Shaban et al., 2017) with a 1-shot setup to test our model, HSDNet, alongside the baseline model, HDMNet. PASCAL $- {5^i}$ is constructed based on the PASCAL VOC 2012 dataset (Everingham et al., 2010) and is enhanced with additional annotations from SDS (Hariharan et al., 2011), encompassing 20 classes. The link to the dataset is PASCAL $- {5^i}$ dataset. We selected the first five classes—namely aeroplane, bicycle, bird, boat, and bottle—as new classes for testing, using the remaining 15 classes as the training set. To ensure the stability and fairness of our experiments, we randomly drew 1,000 query/support pairs for the PASCAL $- {5^i}$ tests. The results are shown in Table 5.

**Table 5:** Results on the PASCAL $- {5^i}$ dataset.

model	Aeroplane	Bicycle	Bird	Boat	Bottle	Avg
HDMNet (Baseline)	**88.64**	37.42	84.17	**68.66**	65.14	68.81
HSDNet (Ours)	87.40	**38.86**	**84.70**	65.46	**69.70**	**69.22**

From Table 5, it is evident that on the PASCAL $- {5^i}$ dataset, our model significantly outperforms the baseline model, with HSDNet’s mIoU being 0.41% higher than that of HDMNet. This demonstrates that although our model is specifically tailored for poultry farming, it also excels on other datasets. While HSDNet competes closely with HDMNet in categories such as aeroplanes and boats, it demonstrates distinct advantages in more complex categories like bicycles, birds, and bottles. These results affirm the effectiveness of HSDNet in handling various challenging segmentation tasks, highlighting its potential for widespread application in semantic segmentation.

**Figure 11:**
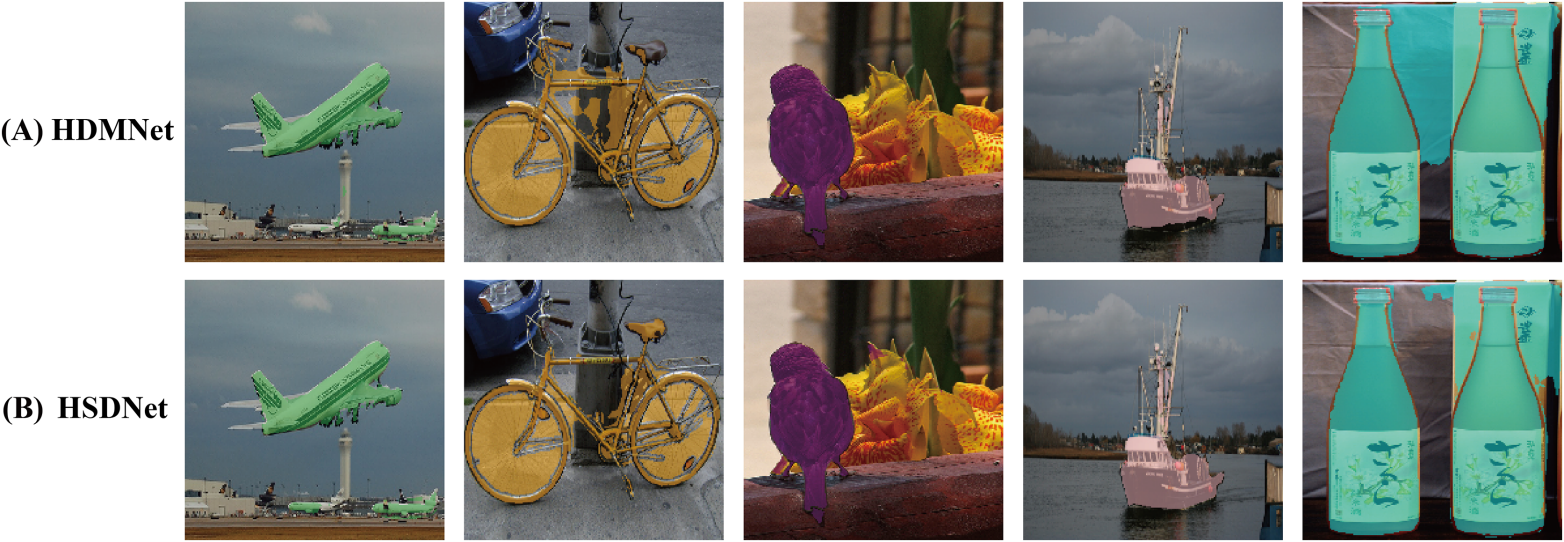
The figure illustrates the visual comparison between two models on the PASCAL $- {5^i}$ dataset. The top row features results from the baseline model HDMNet, and the bottom row displays outcomes from our model, HSDNet. Data sourced from the voc result.

To more effectively showcase our test results, we conducted visualizations, as depicted in Fig. 11. The first row illustrates the outcomes from the baseline model, HDMNet, while the second row shows results from our model, HSDNet. From Fig. 11, it is evident that our model successfully covers three airplanes, compared to the baseline model, which covers only two, indicating our model’s enhanced performance. Moreover, HDMNet sometimes masks regions not pertaining to the actual category, as seen in the second column with the bicycle’s triangular frame and in the fifth column between two bottles, where the predictions are less accurate. In contrast, our HSDNet more effectively differentiates between the background and objects, accurately covering the bicycle’s seat and correctly identifying non-bicycle parts. Additionally, our model achieves more complete coverage of targets, as seen with the boat in the fourth column, where HSDNet more effectively masks the boat, particularly at the boundary between the boat and the water. By testing HSDNet on various objects in different environments, we first demonstrated better segmentation performance for dense scenes or small targets, and second, experiments on other datasets also demonstrated our robustness.

### Parameter analysis

In order to gain a deeper understanding of the model’s performance and stability under different parameter settings, we designed five detailed parameter experiments. Firstly, we explored the base optimizer of SAM, choosing SGD and Adam for experimentation to determine which one is better suited for our model. Subsequently, we tested the rho value in the SAM optimizer, setting it to 0.01, 0.005, and 0.1 respectively. This parameter plays a crucial role in guiding the direction of model parameter updates. Additionally, we investigated the adaptive parameter of SAM, setting it to both True and False to explore the impact of adaptive learning rate adjustments. At the same time, we delved into the role of Nesterov momentum in the model, especially its potential value in accelerating model convergence and enhancing performance. Lastly, we conducted tests on the $\gamma$ parameter in DiceLoss, using three different values: 0.1, 0.5, and 1, aiming to optimize the model’s segmentation performance in imbalanced categories. The results of the parameter experiments are shown in Table 6.

**Table 6:** Parameter analysis was performed on the poultry farming dataset with ResNet50.

Settings	Value	Chicken	Duck	Fish	Avg
SAM-base optimizer	SGD	81.37	67.77	69.54	72.89
	Adam	78.90	64.79	68.30	70.66
SAM-rho	0.01	78.63	65.72	69.28	71.21
	0.05	81.37	67.77	69.54	72.89
	0.1	78.18	62.86	67.74	69.59
SAM-adaptive	False	78.63	65.72	69.28	71.21
	True	81.37	67.77	69.54	72.89
SAM-nesterov	False	79.30	65.62	68.36	71.09
	True	81.37	67.77	69.54	72.89
Loss- $\gamma$	0.2	78.76	64.20	70.34	71.10
	0.3	81.37	67.77	69.54	72.89
	0.4	78.57	65.27	67.80	70.55

As can be seen from Table 6, the best results were achieved when SGD was selected as the base optimizer for SAM, the rho value in SAM optimizer was set to 0.05, the adaptive parameter was set to True, and Nesterov was set to True. For the $\gamma$ parameter in diceloss, a setting around 0.3 yielded the best performance. It’s worth noting that $\gamma$ is not a meticulously chosen value but rather a range. Within this range, good convergence results are observed. Depending on the dataset, there might be some adjustments to this parameter.

## Discussion

### Contribution of the proposed method

Common semantic segmentation techniques rely on large datasets with precise annotations to train models. In the context of poultry farming, it often requires the collection and annotation of thousands of images, covering a variety of poultry breeds, behaviors, and potential breeding environment conditions, to achieve the desired accuracy and generalization capabilities. This necessitates significant upfront costs and time investment. Moreover, when faced with new scenes or changes in the environment, traditional semantic segmentation methods may need to recollect a large amount of data and retrain models, hindering the model’s rapid deployment and dissemination. In contrast, as demonstrated in Table 2, HSDNet requires only one image for training and achieves an mIoU of 72.89%, with the IoU for chickens reaching as high as 81.37%. This alleviates the traditional semantic segmentation’s heavy dependency on large datasets. HSDNet can quickly learn from a few examples, this flexibility allows it to swiftly adapt to new poultry breeds or specific behavioral patterns never encountered before, exhibiting superior generalization capabilities. This showcases HSDNet’s remarkable ability to push the boundaries of semantic segmentation in challenging and data-scarce environments, marking the dawn of a new era for efficient and adaptable machine learning applications in agriculture and beyond.

### Limitations and future work

While HSDNet has shown promising results in poultry farming, its generalization capabilities to other agricultural areas or significantly different environments are not yet fully explored. The performance in more complex or less structured environments may vary. Furthermore, HSDNet’s effectiveness is greatly influenced by the quality of annotations; inaccurate or inconsistent annotations could impact its learning ability and accuracy. The complexity of the method also leads to considerable computational expenses.

In our future work, we will further explore few-shot semantic segmentation models, attempting to incorporate incremental learning into HSDNet. This will allow the model to adapt to new information over time without the need for complete retraining, making it more adaptable to the dynamic environments found in poultry farming with diseases. We will also work on optimizing the model’s structure to improve accuracy while making the model lighter, thus easing its deployment in the field.

## Conclusions

In summary, this study addresses the significant challenges associated with traditional semantic segmentation, including the high demand for annotated datasets and the difficulty in adapting to the complex and diverse environments found in poultry farming. The integration of few-shot learning into poultry farming *via* the HSDNet model significantly enhances the efficiency and effectiveness of AI applications in agriculture. HSDNet is capable of rapidly adapting to new environments or species with minimal data input, achieving a notable semantic segmentation accuracy of 72.89% on single images. The innovative combination of a sharpness-aware model with Dice loss is crucial for addressing typical issues in agricultural settings, such as non-smooth losses and sample imbalances, thereby improving the model’s stability and accuracy.

Crucially, the HSDNet model’s robustness and versatility are further evidenced by its successful application not only in poultry but also in monitoring aquatic species such as the golden crucian carp. This broadens the HSDNet model’s applicability across different agricultural sectors. Our approach effectively diminishes the reliance on extensive data collection and manual annotation, positioning it as a feasible solution for addressing real-world agricultural challenges.

Overall, our findings validate our hypothesis and fulfill the study’s goals by demonstrating that few-shot learning can be effectively adapted for AI-driven agricultural applications. This research paves the way for further exploration and continuous enhancement of AI technology in agriculture, aiming to improve adaptability and operational efficiency across diverse farming environments.

## Additional Information and Declarations

### Data Availability

The following information was supplied regarding data availability: The code is available at GitHub: https://github.com/DaixianLiu/DaixianLiu.github.io. The duck dataset is available at GitHub: https://github.com/DaixianLiu/DaixianLiu.github.io/tree/main/duck. The fish dataset is available at GitHub: https://github.com/DaixianLiu/DaixianLiu.github.io/tree/main/fish. The chicken dataset is available at GitHub: https://github.com/DaixianLiu/DaixianLiu.github.io/tree/main/chicken. The goose dataset is available at GitHub: https://github.com/DaixianLiu/DaixianLiu.github.io/tree/main/goose. The code and duck, fish, chicken and goose datasets are also available at Zenodo: DaixianLiu. (2024). DaixianLiu/DaixianLiu.github.io: first release (v1.0.0). Zenodo. https://doi.org/10.5281/zenodo.11157859. The PASCAL-5i dataset is available at http://host.robots.ox.ac.uk/pascal/VOC/voc2012/.
